# The Effects of Horticultural Activity Program on Vegetable Preference of Elementary School Students

**DOI:** 10.3390/ijerph18158100

**Published:** 2021-07-30

**Authors:** Ha-Ram Kim, Seon-Ok Kim, Sin-Ae Park

**Affiliations:** 1 Department of Animal and Plant Assisted Therapy, Graduate School of Agriculture and Animal Science, Konkuk University, Seoul 05029, Korea; hahaharam123@naver.com; 2 Department of Bio and Healing Convergence, Graduate School, Konkuk University, Seoul 05029, Korea; kso0804@naver.com; 3 Department of Systems Biotechnology, Konkuk Institute of Technology, Konkuk University, Seoul 05029, Korea

**Keywords:** eating habits, horticultural education, mediating factors, nutrition education, parent education, vegetable intake

## Abstract

This study was conducted to investigate effects of a horticultural activity program based on a mediating variable model for improving vegetable preference among elementary students. A quasi-experimental design was employed with 136 students and 136 primary carers in Seoul, South Korea. Based on the mediation model for improving children’s vegetable preference, 12 sessions were conducted, including gardening, nutrition education, and cooking activities using harvests. The program was conducted weekly for 12 weeks from March to July 2019. To investigate the effect of this program, mediating factors of the children were evaluated before and after the program. Pearson correlation analysis was used to identify the mediating factors. The nutrition index, attitude, knowledge, and eating habits of the primary carers were evaluated. Results showed children’s nutrition and gardening knowledge, dietary self-efficacy, outcome expectancies, and vegetable preference were significantly improved (*p* < 0.001). Primary carers showed significant improvement in the nutrition index, knowledge, and attitude (*p* < 0.05). The correlation analysis confirmed that most of the mediating factors had significant correlations (*p* < 0.05). Therefore, administering a structured program involving horticultural activities and nutrition education as mediating factors for 12 sessions was effective in improving eating behavior for vegetables elementary school students and primary carers.

## 1. Introduction

School age is an essential period when eating behavior is formed; unlike infants, children tend to select the foods they want to eat, but this often leads to inappropriate eating behavior such as excessive snacking and unbalanced meals [1]. According to the 2018 Korea health statistics [2], which recorded daily intake per food group, vegetable intake for children aged 6–11 was 148.3 g, which was less than half of the recommended amount. Since eating behavior continues from childhood into adolescence and adulthood [3], increasing fruit and vegetable consumption at an early age is important [4]. During this period of rapid growth, malnutrition leads to delays in physical development and overnutrition leads to obesity [5], which cannot only severely impact children’s development but also result in the deterioration of learning ability, psychological state, and emotional problems [6].

Dietary intake and eating behavior of school-aged children are influenced by school life, mass media, and family life [7]. Notably, nutrition knowledge and attitudes of parents are important factors that determine the nutritional status of their children [8]. Since the role of parents is emphasized in relation to children’s eating behavior and unbalanced diet, it is essential that nutrition education for appropriate eating is administered not only to children but also to their parents [9].

Horticultural activity programs help in acquiring plant-related information and nutrition knowledge, and cultivating a sense of accomplishment, by caring for vegetables [10]. Particularly, horticultural activity programs in schools not only encourage children’s interest in planning and managing their gardens through a given task but also offer an opportunity for them to collaborate with their peers, face challenges efficiently, and develop confidence [11,12,13]. Today, gardening is not just for the purpose of producing crops, but is being promoted as an educational tool that can improve children’s positive attitudes and preferences toward vegetables and fruits [14]. Intervention in horticultural activities can provide benefits such as weight loss and improved nutritional status of children as a result of physical activity, as well as increased intake of vegetables and fruits [15].

Such programs also allow them to make better food choices and recognize the importance of a healthy diet [16]. This process is beneficial in shaping children’s preference for vegetables or at least trying them and it can also successfully increase vegetable consumption [17,18].

Cooking activities utilizing school garden resources are carried out by preparing gardens and harvesting, through which children are exposed to food and their knowledge of and preference for vegetables are improved, resulting in a greater willingness to eat new foods [19,20,21,22]. Previous studies have shown that using school gardens and kitchens to develop students’ healthy eating habits is effective in promoting interest in food preparation, preference for certain food ingredients, and selecting healthy foods [18,23,24,25]. In another study [26], food preference, cooking skills, self-efficacy, knowledge in, and food preparation frequency increased through culinary interventions in schools, which helped foster healthy eating habits.

However, there is a lack of integrated education programs, aimed at improving the vegetable eating behavior of children, that have integrated horticultural activities, nutrition education, and cooking activities, using gardens.

This study extracted mediator factors between horticultural activity and improvement of children’s eating behavior based on related research. After the program was implemented, through correlation analysis between mediating factors, socio-psychological predictive variables affecting children’s vegetable preference were selected. Therefore, this study aims to investigate the effects of an integrated horticultural activity program, related to the improvement of vegetables eating behavior among elementary school students as well as parental nutrition education.

## 2. Materials and Methods

### 2.1. Participants

To recruit participants, an official letter regarding this study was sent to 26 elementary schools in Seoul, South Korea. Of the 26 schools that received the official letter, 6 schools understood the contents of this study and expressed their intention to participate. Finally, the J Elementary School in Seoul, South Korea was selected considering the school’s garden environment, class schedule, and the number of students available to participate in this study. Participants were sent a research-related public relations home correspondence through the school electronic notification service, and we received a consent form from participants and their legal representatives who volunteered to participate. Finally, this study was conducted from March–July 2019 with a total of 136 students and 136 primary carers, consisting of 71 third-grade and 65 fifth-grade students (average age: 11.0 ± 1.0) in the J Elementary School in Seoul, South Korea. Only one primary carer of each participating children participated in this study. The sample size was set at 100 or more, referring to previous studies involving behavioral theory to increase children’s vegetable and fruit intake [19,27].

Before the program was conducted, researchers visited the school and provided detailed explanations and an orientation on the purpose, schedule, and precautions of the study to the participating students. A demographic information questionnaire was completed by all participating students. This study was approved by the Institutional Review Board of Konkuk University (7001355-201903-HR-297).

### 2.2. Developing a 12-Session Horticultural Activity Program

This study included a garden-based horticultural activity program that introduced nutrition education and cooking activities using harvests to improve vegetable consumption behavior of elementary school students. It comprised a 12 horticultural activity program, which were modified versions of the horticultural activity program developed by Kim and Park [28], aimed at improving children’s vegetable eating behavior based on a mediating variable model (Table 1).

Through previous study findings on eating behavior of elementary school students, this program introduced the mediating factors of eating behavior improvement such as horticultural and nutrition knowledge [29,30,31,32], dietary self-efficacy [30,31,32], acquisition of preparation skills [33,34,35], willingness to taste, and preference for vegetables [29,35,36], related to the social cognitive theory. In the social cognitive theory, the dietary self-efficacy is included in the personal determinants, and acquisition of preparation skills and knowledge are included in the behavioral determinants [28]. Additionally, willingness to taste, and preference for vegetables could be enhanced by the environmental determinants such as observational learning and reinforcements. These three determinants could interact with each other and influence changes in eating behavior of children [1].

Crops suitable for school garden activities were selected according to the season and farming cycle, as well as planned gardening activities by referring to the cultivation methods, functions, and characteristics of the crops [37]. The contents of gardening knowledge education mainly focus on the cultivation method, characteristics, and functional contents of each crop, and the program was designed in consistence with the practical curriculum of elementary schools [38]. The contents of nutrition education were adjusted to reflect the difficulty by grade with reference to the food safety and nutrition education textbooks published by the Ministry Food and Drug Administration and the Ministry of Education for middle and high grades [39,40]. Cooking activities were developed taking into account the session time, based on the harvest time of crops grown directly in the garden.

Children’s activity sheets were distributed each session to help participants understand the educational contents, and newsletters including cooking recipes using crops harvested in the program were sent to the families. The newsletter contained information on nutrition and recipes for primary carers and children to experience cooking activities together. At the end of each session, we sent a newsletter to all participants through the school’s electronic notification service so that primary carers could know the vegetables and cooking methods used that day as well as introduced nutrition information for the vegetables. When primary carers and children cooked together and shared their activities with their friends, they talked and praised each other for their activities, which gave them further encouragement.

#### Program Operation

This program was conducted once a week for 80 min per session utilizing three spaces: the regular school class for nutrition education, the rooftop garden (38 m^2^) for horticultural activity, and the practical arts room for cooking activity. The time allocated for each activity was approximately 20 min for nutrition education, 30 min for horticultural activity, and 30 min for cooking activity. For example, the 6th session was carried out under the theme of “Safe Food Choice and Food Poisoning Prevention”. The activities carried out under this theme involved harvesting and cooking of chives (Table 2). The participants were expected to harvest and cook the chives, as a result of which they were able to acquire horticultural and nutrition knowledge about chives, and the skills involved in their preparation (behavior domain). In addition, harvesting and cooking chives grown directly by the students were intended to increase their dietary self-efficacy (personal domain). By talking with friends about their experience of eating vegetables, observational learning and positive behavior reinforcement were induced (environmental aspect). Thus, applying the factors of eating habit improvement and social cognitive theory in the program was intended to increase their vegetable preferences.

### 2.3. Assessments

#### 2.3.1. Mediating Factors Related to Children’s Eating Behavior

The children’s nutrition index was developed by The Korean Nutrition Society and Amway Korea [41], and a total of 22 items updated by The Korean Nutrition Society in 2014–2015 were used. The sub-items are divided into “balance”, “resection”, “diversity”, “practice”, and “environment”, and the scores are categorized into high class (75–100%), medium-high (50–74%), medium-low (25–49%), and lower grades (0–24%). It consists of questions about the frequency and attitude of food intake, and attitude toward daily life, which means that the higher the score, the better the nutritional status.

To assess children’s gardening knowledge, a total of ten selected questions were extracted from the gardening knowledge scale [28] by referring to selected crops and actual gardening activities by experts. The subtopics included plant functions, environmental factors needed for plant growth, kinds of vegetables, and gardening tools, and were scored by giving one point for each question. The total score ranged from 0 to 10 and higher scores were indicative of greater gardening knowledge.

The nutrition knowledge scale was developed by referring to the textbooks produced by the Ministry of Food and Drug Administration and the Ministry of Education for the middle and high elementary school grades [39,40]. The third-grade students were asked 15 questions and the fifth-grade students were asked 21 questions. Each item had three response options: “Yes”, “No”, or “I don’t know”. A correct response was given 1 point, and an incorrect response was given 0 points. Higher scores were indicative of greater nutrition knowledge.

The vegetable preference scale was developed by adding the representative food list of vegetables selected by the 2015 Korean Nutrition Intake Standard [42]. It consists of 29 questions and 5-point Likert scale, and each item had five response options: “greatly dislike”, “hate”, “neutral”, “like”, and “like a lot”. Higher scores were indicative of higher vegetable preference. The total score ranged from 0 to 145.

The outcome expectancy questionnaire on the fruit and vegetable intake developed by Domel et al. [43] was used. The sub-items comprise 17 questions related to the social health, physical ability, and behavioral domains. The three-point Likert scale includes reverse-scored questions, and the higher the total score, the higher the expectation for positive results. The total score of the questionnaire ranged from 0 to 34. In this study, Cronbach’s alpha was 0.63 in both the pre-program and post-program tests. Kim and Park’s [28] study showed that the Cronbach’s alphas were 0.78 and 0.82 in the pre-program and post-program test, respectively.

The questionnaire for dietary self-efficacy developed by Domel et al. [44] based on the social cognitive theory was used after being modified and supplemented for this study. Because fruits are not covered in this program, items related to fruits were deleted from the questionnaire, and only items related to vegetables were selected and used. The questionnaire is assessed on a 3-point Likert scale with 24 items such as whether to buy fruit and vegetable and whether to choose fruit and vegetable at mealtime or snack time; the higher the score, the higher the dietary self-efficacy for vegetables and fruits intake. The total score of the questionnaire ranged from 0 to 48. In this study, the Cronbach’s alphas were 0.60 and 0.61 in the pre- and post-program test, respectively. In the study of Domel et al. [44], Cronbach’s alpha was reported as 0.76, and that in Kim and Park’s study [28] were 0.89 and 0.90 in the pre- and post-program test, respectively.

Food neophobia scale, which translated the food neophobia scale (FNS) developed by Pliner and Hobden [45] into a 3-point Likert scale considering the age of the research subjects, was used. There were a total of ten items, and five of them were reverse-scored questions. Food neophobia is a tendency to reject new food, and the higher the score, the higher the food neophobia. The total score of the questionnaire ranged from 0 to 20. In this study, Cronbach’s alphas were 0.69 and 0.69 in the pre-program and post-program test, respectively. In the study of Kim and Park [28], Cronbach’s alphas were 0.78 and 0.68 in the pre-program and post-program test, respectively.

#### 2.3.2. Evaluation Items Related to Primary Carers’ Eating Behavior

The adult nutrition index was developed by The Korean Nutrition Society in 2018 and consists of 21 items. The sub-items are divided into “balance”, “diversity”, “excision”, and “practice”, and are judged as high (75–100%), middle (25–74%), and low grades (0–24%). The questions related to the frequency of food intake, attitude toward eating behavior, and attitude toward daily life were included, which means that the higher the score, the better the nutritional status.

The nutrition attitude scale developed by Lee et al. [46] consisted of five questions including interest in nutrition, experience of nutrition education, medium of nutrition education acquisition, delivery of nutrition knowledge, and existence of life practice of nutrition knowledge. Each item had three response options: “No”, “Neutral”, and “Yes”. Higher scores were indicative of greater attitude of nutrition.

The nutrition knowledge scale was developed by referring to textbooks produced by the Ministry of Food and Drug Administration and the Ministry of Education for the middle and high grades [39,40] of elementary school and the contents of newsletters distributed in the program. Each item had three response options: “Yes”, “No”, or “I don’t know”. A correct response was given 1 point, and an incorrect response was given 0 points. There were a total of 20 items and the total score of the scale ranged from 0 to 20. Higher scores indicated greater knowledge of nutrition.

The eating habit scale was developed by Lee et al. [46] and included 21 questions including breakfast frequency, meal amount, meal speed, unbalanced eating, snack frequency, eating frequency with family, and eating habit problems. Of these, only Questions 1, 2, 3, 4, and 6 were evaluated and scored. The selected items are related to the nutrition education contents in the newsletter sent home. The total score of the questionnaire ranged from 0 to 15. The higher the score, the better the eating behavior.

#### 2.3.3. Satisfaction Survey

The researchers revised the questionnaires developed by Park et al. [47] to investigate participants’ satisfaction with the horticultural activity program. The satisfaction questionnaire consisted of six items: overall satisfaction, satisfaction with program time, satisfaction with program frequency, preferred activities, willingness to re-participation, and the possibility of recommending the program to others. In addition, the questionnaire for primary carers and teachers developed by Kim and Park [28] consisted of items that evaluated overall satisfaction, satisfaction with education and newsletter contents, willingness to re-participation, possibility by recommending programs to others, and whether children’s eating behavior improved after the program.

### 2.4. Data Analysis

A total of 136 elementary school students (third grade: 71 students, fifth grade: 65 students) and 136 primary carers participated in the experiment. The results of the study were analyzed, with the exception of unreturned questionnaires from primary carers. The statistical package for the social sciences (SPSS) version 25 for Windows (IBM, Armonk, NY, USA) was used to analyze the evaluation items of the mediating factors that improve eating behavior before and after the horticultural activity program and Cronbach’s alpha coefficients. The evaluation items related to eating behavior compared with the pre-score and post-score were analyzed using the paired sample t-test. The correlation between the factors of eating behavior was analyzed by obtaining Pearson’s correlation coefficient. The results of the satisfaction survey were analyzed using MS Excel (Office 2016; Microsoft Crop., Redmond, WA, USA) and multiple response analysis was conducted. The statistical significance level was based on *p* < 0.05.

## 3. Results

### 3.1. Participant Characteristics

The characteristics of the experimental group children and primary carers who participated in this program are as follows (see Table 3 and Table 4). Among the 136 children who participated in the program, 71 and 65 were in the third and fifth grade, respectively. Among the 136 primary carers who participated, 122 primary carers excluding the missing data were used for data analysis, with 61 primary carers for both third and fifth grade students.

### 3.2. Changes in Mediating Factors Related to Children’s Vegetable Eating Behavior

After completion of the 12 sessions of the horticultural activity program, participants’ gardening knowledge, nutrition knowledge, outcome expectancies, dietary self-efficacy, and vegetable preference were found to have significantly improved (*p* < 0.05; Table 5). Comparing by grade, the third graders’ gardening knowledge, nutrition knowledge, outcome expectancies, dietary self-efficacy, and vegetable preference improved significantly (*p* < 0.05; Table S1); for fifth graders, gardening knowledge, nutrition knowledge, and vegetable preference were significantly improved (*p* < 0.05; Table S2).

There was no significant difference in the children’s nutrition index score before and after the program. However, in the sub-items of the children’s nutrition index, “resection” score lowered significantly, and the “practice” score improved significantly (*p* < 0.05).

In contrast, a comparison of food neophobia scores before and after the program revealed no significant difference for all students (*p* > 0.05).

### 3.3. Correlation between Changes in Mediating Factors Related to Vegetable Eating Behavior

**Figure 1:**
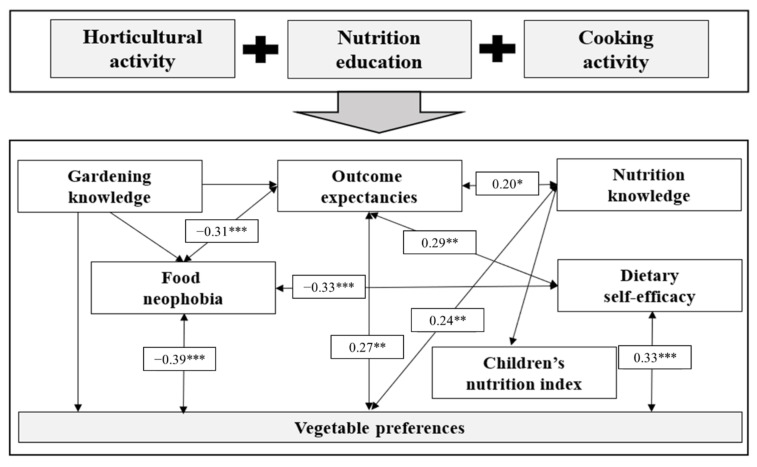
The result of the correlations between mediating factors for improving elementary school children’s vegetable preference (*N* = 136). * *p* < 0.05, ** *p* < 0.01, *** *p* < 0.001. A correlation coefficient of less than ±0.2 means little correlation, that of ±0.2 to ±0.4 means low correlation, that of ±0.4 to ±0.7 means somewhat high correlation, and that of more than ±0.7 means high correlation [48].

After the program, there were significant correlations between the factors related to improving eating behavior. Nutrition knowledge had positive correlations with outcome expectancies (correlation coefficient: 0.20) and vegetable preference (correlation coefficient: 0.24), and outcome expectancies had a positive correlation with vegetable preference (correlation coefficient: 0.27). Dietary self-efficacy had positive correlations with outcome expectancies (correlation coefficient: 0.29) and vegetable preference (correlation coefficient: 0.33). Food neophobia showed negative correlations with dietary self-efficacy (correlation coefficient: −0.33), outcome expectancies (correlation coefficient: −0.31), and vegetable preference (correlation coefficient: −0.39) (Figure 1).

### 3.4. Changes Related to Primary Carers’ Eating Behavior

After the 12-session horticultural activity program, the primary carers’ adult nutrition index, nutrition attitude score, and nutrition knowledge score improved significantly (*p* < 0.05; Table 6). However, there was no significant difference in primary carers’ eating habit scores (*p* > 0.05) before and after the program.

### 3.5. Satisfaction with the Program

**Figure 2:**
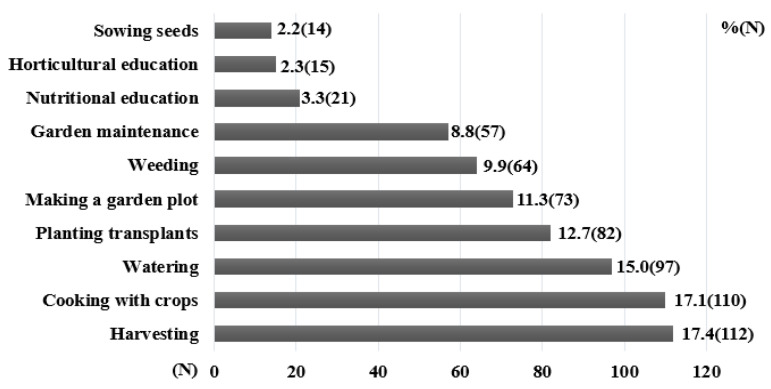
Activity preferences in children in a 12-session horticultural activity program.

The result of the satisfaction survey was that 97.7% of 136 students who participated in the program answered that they were satisfied with the overall program. Moreover, 94.5% of the participants said that they would participate in the program again, and 92.2% of the participants said that they would like to recommend the program to their friends. The preferred activities for participants were harvesting (17.4%), cooking activities using harvested vegetables (17.1%), and watering (15.0%) (Figure 2). According to the results of the teacher satisfaction survey, all teachers (six in total) were very satisfied with the overall program and answered that there was a positive change in the eating behavior of the students that participated in the study. In addition, 76.0% of the primary carers (122 in total) answered that they were satisfied with the overall program, and 88.8% answered that there was a positive change in their children’s eating behavior.

## 4. Discussion

This study examined whether a horticultural activity program for improving the vegetable preference of elementary school students had a positive effect on the eating behavior of children and their primary carers and confirmed the correlations between the mediating factors related to the improvement of eating behavior. Results demonstrated that a program combining nutrition education based on horticultural activities for 12 sessions was effective in improving elementary school students’ vegetable preference and primary carers’ nutritional knowledge, and there were significant correlations between mediating factors for improving children’s eating habits (Figure 1, Table 5 and Table 6).

When comparing differences in scores before and after the horticultural activity program, gardening knowledge, nutrition knowledge, outcome expectancies, dietary self-efficacy, and vegetable preferences of children were significantly improved after the program (Table 5). Previous studies indicated that participation in and experience of a gardening activity program, including nutrition education, improved the nutrition knowledge, gardening knowledge, and preference for vegetables and fruits of elementary students [28,49,50]. The process of planting, cultivating, harvesting, and cooking plants through the horticultural activity program was an effective way to increase knowledge of horticulture and vegetable consumption [24]. Improved nutrition and gardening knowledge could improve behavioral capability for vegetable consumption by helping children make better food choices and recognize the importance of a healthy diet [16,51]. Additionally, a high preference for vegetables and fruits is a major predictor of children’s vegetable and fruit intake [52,53], which is thought to have been increased through direct cultivation opportunities in this program. In addition, students were able to gain positive experience of harvesting and preparing vegetables with their peers, which is judged to have a positive effect on increase their behavioral ability for vegetable consumption.

Dietary self-efficacy means confidence or expectation regarding an individual’s ability to perform a series of behavioral processes to achieve certain results [41,54]; the higher it is, the healthier the food that will likely be chosen, and more vegetables and fruits that will be consumed. Dietary self-efficacy is developed through direct experience, observation, praise, and persuasion [55]. In this study, it is believed that dietary self-efficacy has improved through direct experiences of growing vegetables and eating with friends and primary carers, and the improved self-efficacy provided a sense of pride in eating vegetables.

Outcome expectancies refer to the positive or negative perception of the outcome of a particular behavior, and the stronger the positive perception, the greater the motivation to achieve the behavioral goal [55,56]. O’Brien and Shoemaker [30] showed that an after-school program including eight weeks of gardening and nutrition education increased self-efficacy and improved outcome expectancy of gardening activities of the experimental group compared to the control group. In this study, it is thought that the nutritional benefits of various vegetables as children participated in nutrition education and the enjoyment and sense of achievement of eating vegetables by cooking themselves increased their positive outcomes expectancies.

Participation in the horticultural activity program resulted in no significant difference in the children’s nutrition index and food neophobia scores (Table 5). The children’s nutrition index can evaluate their eating behavior and nutritional status, and it was confirmed that scores, both before and after this program, remained at the mid-level grade. In the sub-items, the “resection” score lowered, and the “practice” score improved significantly after the program. It means that, even after the program, students ate unhealthy foods and snacks, but they checked the nutrition labeling when purchasing food and made efforts such as handwashing before eating, as they learned from nutrition education. Since the nutrition index of children cannot be changed in a short period of time, effective and steady intervention is needed to gradually increase students’ nutrition index to a level indicative of good nutritional status.

Food neophobia means avoiding and/or feeling disgust with new foods, which can be reduced by increasing familiarity with novel or unfamiliar foods [57]. Moreover, repeated exposure to new foods and vegetables is an effective strategy to reduce food neophobia in children [58,59]. Human behavior modification does not occur in a short period of time, but changes slowly as cognitive, behavioral, and environmental factors interact with each other [51]. Therefore, it is necessary to carry out a garden-based integrated program for a longer time to potentially improve students’ willingness to experience and taste vegetables.

As a result of correlation analysis between the mediating factors of eating behavior improvement, all factors except gardening knowledge showed significant correlations (Figure 1). It is judged that children felt a sense of accomplishment by growing and managing vegetables directly in this study and it made them feel familiar with eating vegetables, reduced food neophobia, and increased dietary self-efficacy, outcome expectancies, and vegetable preferences. Similarly, it was reported that outcome expectancies showed positive correlations between dietary self-efficacy, vegetable preference, vegetable and fruit knowledge, and consumption [44,60]. In this study, similar to the results of previous studies [28,56], dietary self-efficacy showed positive correlations with outcome expectancy and vegetable preference. The horticultural activity program in this study, which appeared to have close correlations between mediating factors of improvement of eating behavior, enabled students to improve various mediating factors related to eating behavior through direct experience and improved vegetable preference finally, which are thought to increase vegetable consumption.

After the program, scores for primary carers’ nutrition index, nutrition attitude, and nutrition knowledge improved significantly (Table 6). The increase in nutrition attitude means that the interest in nutrition has increased and the nutrition knowledge is being practiced in daily life. According to Lee’s study [61], the higher the mother’s nutritional attitude score, the higher the correlation between mother’s nutrition knowledge score and the child’s nutrition knowledge score. It is believed that the newsletter, which covered children’s daily horticultural activities, cooking activities, and cooking recipe of vegetables, increased primary carers’ interest and nutrition attitude. In addition, the total nutrition index and the sub-item “resection” improved significantly. It means that primary carers tried to avoid giving their children unhealthy food such as instant noodles, snacks, sweet and oily breads, sweetened beverages, and fast food after the program.

Mothers with excellent nutrition knowledge showed higher awareness regarding the importance of guidance in eating behavior and prepared healthier meals at home than mothers with low nutrition knowledge [7]. Additionally, primary carers who regularly received newsletters and homework for primary carers and children, could increase the effectiveness of the gardening program such as dietary behavior and health outcomes [62,63,64]. Through the horticultural activity program in this study, it was confirmed that indirect nutrition education through newsletters was effective in improving primary carers’ nutrition knowledge by encouraging them to participate in cooking activities with their children.

As a result of the horticultural activity program, there was no significant difference in the primary carers’ eating habits score (Table 6). After the program, nutritional attitude, knowledge, and index were significantly improved, but there was no change in eating habits. In the case of adults, the change in eating habits is not readily apparent even when other related factors have been improved, since eating habits are already formed [1]. Additionally, it is judged that various factors such as primary carers’ academic background and income influence primary carers’ eating habits [65,66]. In the future, it is necessary to consider various factors affecting the eating habits of the primary carers in the program.

In previous studies to increase vegetable and fruit consumption of children and adolescents, the consumption of fruits and vegetables was higher in participants who participated in various interventions, such as cooking and gardening activities, compared to participated only in nutrition education [26,34]. This means that an integrated education program consisting of horticultural activity, nutrition education, and cooking activity can be more effective than an education program that emphasizes only a single intervention. Previous studies have shown that when children share or participate in cultivation and cooking processes at home, primary carers are encouraged to increase vegetable and fruit consumption, buy vegetables and fruits at any time, and make healthy foods [24,67,68]. If the joint efforts by the school and primary carers increase outcome expectancies and dietary self-efficacy and reduce food neophobia, they will exert greater influence on the mediating factors and be more effective in improving elementary school students’ eating behavior.

## 5. Conclusions

The integrated program that combined horticultural activities, nutrition education, and cooking activities for a total of 12 sessions based on mediating factors was found to be effective in improving the vegetable preference of elementary students. It is judged that an attempt to integrate horticultural activities, nutrition education, and parent education that provides an experience of growing vegetables directly played an important role in bringing about a positive effect. This program is meaningful because it involved participation from not only the children but also primary carers who directly affect the children’s eating behavior. It is necessary to create a healthier family by effectively utilizing the added value of families and communities and actively participating and sharing experiences in healthy eating with children and primary carers. It is expected that the horticultural activity program for improving the eating behavior of elementary school students will be continuously expanded and applied through further screening and verification of the mediating factors between horticultural activities and the improvement of children’s eating behavior.

## Figures and Tables

**Table 1:** Development of the horticultural activity program for improving children’s eating behavior for vegetables (modified version of the program developed by Kim and Park [28]).

Session	Program Title	Horticultural Activities	Horticultural Crops	Nutrition Education and Cooking Activity	
				3rd Grade	5th Grade
1	Becoming a child city farmer	Making garden plots Sowing seeds	Potato (Solanum tuberosum) Carrot (Daucus carota)	Six food groups classification Problems of unbalanced diets Benefits of eating balanced diets	
2	Five-colored vegetables that are pleasant to the eyes and mouth	Planting seedlings Sowing seeds	Chives (*Alium tuberosum*) Red radish (*Raphanus sativus*)	Evaluating my eating behaviors Classification of five-colored vegetables and nutrition value How to eat a variety of vegetables	
3	I am a seed planting master	Pulling weeds Sowing seeds	Red beet (*Red rhubarb chard*) Pea (*Pisum sativum*)	The importance of a regular diet The importance of breakfast The importance of regular eating habit	
4	As vegetables grow, we also grow	Planting seedlings	Lettuce (*Lactuca sativa*)	Recommended daily vegetable intake How to eat enough vegetables	
5	Secrets of healthy vegetables	Planting seedlings Setting up plant stakes Fertilizing	Tomato (*Solanum lycopersicum*) Eggplant (*Solanum melongena*)	Nutrients necessary for our body Major nutrients for each food group Solving various nutrients quiz	
6	From garden to table: 1	Harvesting Covering roots with soil	Chives (*Alium tuberosum*)	Cooking: Bacon roll with chives	
				Three methods for preventing food poisoning How to choose safe and hygienic food	Three methods for preventing food poisoning How to choose safe and hygienic food How to store food safely
7	From garden to table: 2	Harvesting	Red radish (*Raphanus sativus*)	Cooking: Red radishes sandwich	
				Meaning of processed food Natural food and processed food Function and content of nutrition labelling	Nutritional labeling items of processed foods High calorific and low nutritional foods Information related to food additives
8	From garden to table: 3	Harvesting	Lettuce (*Lactuca sativa*)	Cooking: Lettuce-wrapped tofu	
				Why a low-salt diet is important The role of sodium How to have a low-salt diet	The role of sodium Daily recommended intake of sodium How to have a low-salt diet
9	From garden to table: 4	Harvesting	Red beet (*Red rhubarb chard*)	Cooking: Red beet salad	
				High and low-fat snacks A healthy snack	The kind of snacks we usually eat A healthy snack How to choose a healthy snack
10	From garden to table: 5	Harvesting	Carrot (*Daucus carota*)	Cooking: Carrot canape	
				Sodium overdose problem Foods that help the body excrete sodium	Sodium overdose problem Vegetables that excrete sodium Foods that help the body excrete sodium
11	From garden to table: 6	Harvesting	Tomato (*Solanum lycopersicum*) Eggplant (*Solanum melongena*) Pea (*Pisum sativum*)	Cooking: Five-colored vegetable pizza	
				Function of five-colored vegetables How to eat five-colored vegetables for your health	Function of five-colored vegetables How to eat five-colored vegetables for your health How to choose healthy vegetables
12	From garden to table: 7	Harvesting	Potato (*Solanum tuberosum*)	Cooking: Cheese and potato pancake	
				Integrated nutrition education	

**Table 2:** Example of a session of the horticultural activity program (6th session).

Activity	From Garden to Table	Session	6
**Related unit**	6th grade Practical Arts 02–04: Characteristics of Food and Taste of Food		
**Social cognitive** **related factors**	Hands-on experience for gardening and preparation, Gardening knowledge, Nutrition knowledge, Preparation skills, Willingness to taste, Expectations for results, Self-Efficacy		
**Horticultural activity**	Harvesting chives		
**Nutrition education**	Middle grade < unit 7 > Choose a safe food		
**Cooking activities**	Making of the bacon roll with chives		
**Goal**	1. Nutrition Education - Know what causes food poisoning - Know the three points to prevent food poisoning - Choose safe and hygienic foods	2. Horticultural activities and gardening knowledge - Know how to harvest chives - Know the meaning of covering roots with soil and try it - Know and practice how to cook harvested chives	
**Preparation material**	Scissors, bacon, enoki mushroom, frying pan, cutting board, knife, dish, sanitary gloves, kitchen towels, vinyl, apron, first aid kit, wet tissue		
**Activity order**	**Activity contents**	**Intervention and precautions**	
**Introduction** **(10 min)**	Sharing greetings and talk about past activities Introducing the activities of today	- Raising expectations for harvesting and cooking activities	
**Development 1** **(20 min):** **Horticultural activities**	Talking about the process of harvesting chives Knowing when and how to harvest chives Telling what covering roots with soil is and trying it on carrots and potatoes	- Beware of safety when using horticultural tools - When harvesting chives, point with fingers to tell children where to cut	
**Development 2** **(10 min):** **Nutrition education**	Talking about food poisoning Learning how to choose safe and hygienic food	- Confirm the food sell-by dates and precautions - Introduce the nutritional ingredients of chives	
**Development 3** **(30 min):** **Cooking activities**	Handwashing after putting on an apron Check the sell-by dates and packaging status of the prepared foods Wash the harvested chives and enoki mushroom and cut them into 5 cm lengths Roll by putting chives and enoki mushroom on bacon Cook the bacon roll wrapped chives in a frying pan Tasting food made from vegetables that harvested	- Beware of safety when using cooking tools - Notify children to cut chives and enoki mushroom to one finger length	
**Arrangement** **(10 min)**	Cleaning up the tools and food ingredients used Express the taste of the foods and feelings Introduce the next activity Share photos of children performing cooking activities by referring to newsletters at home; praise and encourage all children to participate Sharing the final greetings		

**Table 3:** Descriptive characteristics of the participating children (*N* = 136).

Variable	3rd Grade (*n* = 71)	5th Grade (*n* = 65)	Total (*N* = 136)
	Mean ± SD ^1^		
Age (years)	10.0 ± 0.0	12.0 ± 0.0	11.0 ± 1.0
	*n* (%)		
Male	35 (49.3)	29 (44.6)	64 (47.1)
Female	36 (50.7)	36 (55.4)	72 (52.9)

^1^ SD: standard deviation.

**Table 4:** Descriptive characteristics of the participating primary carers (*N* = 122).

Variable	Primary Carer of 3rd Grade (*n* = 61)	Primary Carer of 5th Grade (*n* = 61)	Total (*N* = 122)
Age (years)	*n* (%)		
30–35 years old	2 (3.3)	0 (0.0)	2 (1.6)
36–40 years old	23 (37.7)	13 (21.3)	36 (29.5)
41–45 years old	29 (47.5)	33 (54.0)	62 (50.8)
Over 46 years old	7 (11.5)	15 (24.6)	22 (18.0)
Participant	*n* (%)		
Father	8 (13.1)	9 (14.8)	17 (13.9)
Mother	53 (86.9)	51 (83.6)	104 (85.2)
Grandmother	0 (0.0)	1 (1.6)	1 (0.8)

**Table 5:** Comparison of mediating factors related to eating behavior in children before and after the 12-session horticultural activity program (*N* = 136).

Variable	Pre-Test	Post-Test	Significance ^2^
	Mean ± SD ^1^		
Total nutrition index (*n* = 120)	63.24 ± 9.45	63.75 ± 9.72	0.457 ^NS^
Sub-item “Balance”	54.70 ± 14.26	55.28 ± 13.55	0.661 ^NS^
Sub-item “Resection”	62.01 ± 13.79	59.05 ± 16.45	0.024 *
Sub-item “Diversity”	62.81 ± 19.26	63.54 ± 18.51	0.651 ^NS^
Sub-item “Practice”	66.65 ± 17.00	70.11 ± 17.74	0.018 *
Sub-item “Environment”	75.45 ± 18.46	77.49 ± 18.71	0.193 ^NS^
Gardening knowledge (*n* = 128)	2.48 ± 1.40	5.13 ± 2.12	0.000 ***
Nutrition knowledge (*n* = 128)	8.94 ± 4.20	12.26 ± 4.67	0.000 ***
Vegetable preferences (*n* = 122)	88.58 ± 23.52	97.58 ± 23.04	0.000 ***
Outcome expectancies (*n* = 127)	27.06 ± 4.63	28.56 ± 4.03	0.000 ***
Dietary self-efficacy (*n* = 125)	36.94 ± 7.20	39.14 ± 6.75	0.000 ***
Food neophobia (*n* = 127)	6.71 ± 4.20	6.72 ± 4.82	0.964 ^NS^

^1^ SD: standard deviation. ^2^ * *p* < 0.05, *** *p* < 0.001, NS = Non-significant by paired sample *t*-test.

**Table 6:** Comparison of factors related to eating behavior in primary carers before and after the 12-session horticultural activity program (*N* = 122).

Variable	Pre-Test	Post-Test	Significance ^2^
	Mean ± SD ^1^		
Total nutrition index (*n* = 104)	36.77 ± 7.40	38.35 ± 7.15	0.027 *
Sub-item “Balance”	33.58 ± 14.84	33.52 ± 12.78	0.952 ^NS^
Sub-item “Diversity”	42.42 ± 14.55	43.02 ± 14.61	0.708 ^NS^
Sub-item “Resection”	27.84 ± 11.56	31.34 ± 10.43	0.007 **
Sub-item “Practice”	47.11 ±11.91	49.14 ± 11.89	0.058 ^NS^
Nutrition attitude (*n* = 115)	8.64 ± 1.62	9.46 ± 2.10	0.000 ***
Nutrition knowledge (*n* = 118)	11.57 ± 2.60	12.19 ± 2.84	0.003 **
Eating habits (*n* = 97)	10.85 ± 1.71	10.80 ± 1.62	0.745 ^NS^

^1^ SD: standard deviation. ^2^ * *p* < 0.05, ** *p* < 0.01, *** *p* < 0.001, NS = Non-significant by paired sample *t*-test.

## Data Availability

The datasets generated for this study are available on request to the corresponding author.

## Supplementary Materials

These are available online at https://www.mdpi.com/article/10.3390/ijerph18158100/s1, as follows: Table S1: Comparison mediating factors related to eating behavior of the third-grade students before and after the 12-session horticultural activity program (*N* = 71). Table S2: Comparison mediating factors related to eating behavior of the fifth-grade students before and after the 12-session horticultural activity program (*N* = 61).
