# Supplementary material for: The Effects of Horticultural Activity Program on Vegetable Preference of Elementary School Students
Source: Int J Environ Res Public Health. 2021 Jul 30;18(15):8100. doi: 10.3390/ijerph18158100 (PMC8345950; doi:10.3390/ijerph18158100)
Supplement: Supplementary file 1 [file ijerph-18-08100-s001.zip › ijerph-1280830-supplementary.pdf]

Supplemental Tables

Table S1. Comparison mediating factors related to eating behavior of the third-grade students before and after the 12-session horticultural activity program (*N* = 71).

**Variable**	**Pre-test**	**Post-test**	**Significance** [2]
	**Mean ± SD** [1]		
Total Children's nutrition index (*n* = 60)	61.36 ± 9.94	61.60 ± 10.05	0.835 NS
Sub-item "Balance"	52.59 ± 14.26	53.01 ± 14.05	0.844 NS
Sub-item "Resection"	62.45 ± 15.21	59.05 ± 16.92	0.082 NS
Sub-item "Diversity"	60.29 ± 19.17	60.04 ± 19.83	0.923 NS
Sub-item "Practice"	64.88 ± 18.27	68.38 ± 18.08	0.129 NS
Sub-item "Environment"	70.57 ± 19.82	72.70 ± 18.72	0.366 NS
Gardening knowledge (*n* = 65)	2.11 ± 1.48	4.75 ± 2.29	0.000 ***
Nutrition knowledge (*n* = 65)	7.11 ± 3.35	9.57 ± 2.74	0.000 ***
Vegetable preferences (*n* = 61)	82.16 ± 23.38	95.03 ± 24.92	0.000 ***
Outcome expectancies (*n* = 64)	25.75 ± 4.76	27.84 ± 4.34	0.000 ***
Dietary self-efficacy (*n* = 62)	34.90 ± 7.08	38.63 ± 6.56	0.000 ***
Food neophobia (*n* = 64)	7.50 ± 3.69	7.06 ± 4.52	0.403 NS

[1] SD: standard deviation. [2] *$p < 0.05$, **$p < 0.01$, ***$p < 0.001$ by paired sample t-test.

Table S2. Comparison mediating factors related to eating behavior of the fifth-grade students before and after the 12-session horticultural activity program ($N = 61$).

Variable	Pre-test	Post-test	Significance [2]
	Mean ± SD [1]		
Total Children's nutrition index ($n = 60$)	65.13 ± 8.62	65.91 ± 8.95	0.320 NS
Sub-item "Balance"	56.82 ± 14.06	57.55 ± 12.75	0.642 NS
Sub-item "Resection"	61.57 ± 12.32	59.05 ± 16.11	0.157 NS
Sub-item "Diversity"	65.34 ± 19.18	67.04 ± 16.53	0.395 NS
Sub-item "Practice"	68.41 ± 15.60	71.85 ± 17.37	0.065 NS
Sub-item "Environment"	80.33 ± 15.69	82.27 ± 17.59	0.351 NS
Gardening knowledge ($n = 63$)	2.87 ± 1.21	5.52 ± 1.87	0.000 ***
Nutrition knowledge ($n = 63$)	10.83 ± 4.18	15.03 ± 4.65	0.000 ***
Vegetable preferences ($n = 61$)	95.00 ± 21.97	100.13 ± 20.89	0.016 *
Outcome expectancies ($n = 63$)	28.38 ± 4.13	29.29 ± 3.58	0.054 NS
Dietary self-efficacy ($n = 63$)	38.94 ± 6.80	39.63 ± 6.95	0.268 NS
Food neophobia ($n = 63$)	5.90 ± 4.56	6.38 ± 5.12	0.295 NS

[1] SD: standard deviation. [2] *$p < 0.05$, **$p < 0.01$, ***$p < 0.001$ by paired sample t-test.
